# Supplementary material for: Molecular basis of ubiquitin-specific protease 8 autoinhibition by the WW-like domain
Source: Commun Biol. 2021 Nov 8;4:1272. doi: 10.1038/s42003-021-02802-x (PMC8576004; doi:10.1038/s42003-021-02802-x)
Supplement: Supplementary file 3 — Reporting Summary [file 42003_2021_2802_MOESM3_ESM.pdf]

nature portfolio

Corresponding author(s): Toshiaki Fukushima, Masayuki Komada

Last updated by author(s): Sep 16, 2021

Reporting Summary

Nature Portfolio wishes to improve the reproducibility of the work that we publish. This form provides structure for consistency and transparency in reporting. For further information on Nature Portfolio policies, see our Editorial Policies and the Editorial Policy Checklist.

Statistics

For all statistical analyses, confirm that the following items are present in the figure legend, table legend, main text, or Methods section.

n/a	Confirmed	
☐	☒	The exact sample size (n) for each experimental group/condition, given as a discrete number and unit of measurement
☐	☒	A statement on whether measurements were taken from distinct samples or whether the same sample was measured repeatedly
☐	☒	The statistical test(s) used AND whether they are one- or two-sided<br>*Only common tests should be described solely by name; describe more complex techniques in the Methods section.*
☐	☒	A description of all covariates tested
☐	☒	A description of any assumptions or corrections, such as tests of normality and adjustment for multiple comparisons
☐	☒	A full description of the statistical parameters including central tendency (e.g. means) or other basic estimates (e.g. regression coefficient) AND variation (e.g. standard deviation) or associated estimates of uncertainty (e.g. confidence intervals)
☐	☒	For null hypothesis testing, the test statistic (e.g. F, t, r) with confidence intervals, effect sizes, degrees of freedom and P value noted<br>*Give P values as exact values whenever suitable.*
☒	☐	For Bayesian analysis, information on the choice of priors and Markov chain Monte Carlo settings
☒	☐	For hierarchical and complex designs, identification of the appropriate level for tests and full reporting of outcomes
☒	☐	Estimates of effect sizes (e.g. Cohen's d, Pearson's r), indicating how they were calculated

Our web collection on statistics for biologists contains articles on many of the points above.

Software and code

Policy information about availability of computer code

Data collection	No software was used.
Data analysis	Graph Pad Prism 9 and MS Excel 16.48 were used to make graphs and perform statistical analyses.

For manuscripts utilizing custom algorithms or software that are central to the research but not yet described in published literature, software must be made available to editors and reviewers. We strongly encourage code deposition in a community repository (e.g. GitHub). See the Nature Portfolio guidelines for submitting code & software for further information.

Data

Policy information about availability of data

All manuscripts must include a data availability statement. This statement should provide the following information, where applicable:
- Accession codes, unique identifiers, or web links for publicly available datasets
- A description of any restrictions on data availability
- For clinical datasets or third party data, please ensure that the statement adheres to our policy

Data supporting the conclusions of this paper are available from the corresponding authors upon reasonable request and have been included as supplementary materials with this publication.

Field-specific reporting

Please select the one below that is the best fit for your research. If you are not sure, read the appropriate sections before making your selection.

☒ Life sciences ☐ Behavioural & social sciences ☐ Ecological, evolutionary & environmental sciences

For a reference copy of the document with all sections, see nature.com/documents/nr-reporting-summary-flat.pdf

Life sciences study design

All studies must disclose on these points even when the disclosure is negative.

Sample size	All sample sizes of our biochemical and cell biological experiments were predetermined as "three".
Data exclusions	No data were excluded from the analyses.
Replication	All attempts at replication were successful.
Randomization	Allocation was random.
Blinding	No blinding was done during data collection and analysis.

Reporting for specific materials, systems and methods

We require information from authors about some types of materials, experimental systems and methods used in many studies. Here, indicate whether each material, system or method listed is relevant to your study. If you are not sure if a list item applies to your research, read the appropriate section before selecting a response.

Materials & experimental systems

n/a	Involved in the study	
☐	☒	Antibodies
☐	☒	Eukaryotic cell lines
☒	☐	Palaeontology and archaeology
☒	☐	Animals and other organisms
☒	☐	Human research participants
☒	☐	Clinical data
☒	☐	Dual use research of concern

Methods

n/a	Involved in the study	
☒	☐	ChIP-seq
☒	☐	Flow cytometry
☒	☐	MRI-based neuroimaging

Antibodies

Antibodies used	anti-FLAG antibody (Wako, #012-22384, clone 1E6, lot WDL0641), anti-FLAG antibody (Sigma Aldrich, #F1804, clone M2, lot SLBN2445V), anti-Ubiquitin antibody (MBL, #D058-3, clone FK2, lot 04), anti-Ubiquitin antibody (Cell Signaling, #3936, clone P4D1, lot 13), anti-EGFR antibody (MBL, #MI-12-1, clone 6F1, lot 035), anti-α-tubulin antibody (Wako, # 011-25034, clone 10G10), anti-HA antibody (Sigma Aldrich, #12158167001 ,clone 3F10, lot 42155800), anti-HA antibody (Santa Cruz, #SC-53516, clone 153), anti-Myc antibody (Santa Cruz, #SC-40, clone 9E10, lot D0419), anti-Myc antibody (hybridoma supernatant, clone 9E10), anti-GFP antibody (Thermo Scientific, #A-11122, polyclonal, lot 1691382), anti-GFP antibody (MBL, #M-048-3, clone 1E4 , lot 066), anti-14-3-3 antibody (Santa Cruz, #SC-1657, clone H-8, lot C2718), anti-USP8 antibody (homemade, rabbit antiserum), anti-HRS antibody (homemade, rabbit antiserum), anti-STAM1 antibody (homemade, rabbit antiserum).
Validation	The species and applications of each antibodies were validated by the manufacture. Anti-USP8 antibody, anti-HRS antibody, and anti-STAM1 antibody were validated in Kato, M. et al. (2000), Komada, M. et al. (1995), and Mizuno, E. et al. (2004), respectively.

Eukaryotic cell lines

Policy information about cell lines

Cell line source(s)	HEK293 cells, HEK293T cells, and HeLa cells were obtained from ATCC.
Authentication	Cell lines that we used were identified by their morphology.
Mycoplasma contamination	All cell lines tested negative for mycoplasma contamination.
Commonly misidentified lines (See ICLAC register)	n/a
