# Supplementary material for: Molecular basis of ubiquitin-specific protease 8 autoinhibition by the WW-like domain
Source: Commun Biol. 2021 Nov 8;4:1272. doi: 10.1038/s42003-021-02802-x (PMC8576004; doi:10.1038/s42003-021-02802-x)
Supplement: Supplementary file 5 — Description of Additional Supplementary Files [file 42003_2021_2802_MOESM5_ESM.pdf]

Description of Additional Supplementary Files

File name: Supplementary Data 1.
Description: Source data underlying the graphs presented in the article, including Figures 1e, 2d, 2e, 3f, 3g, 3i, 4b, 4c, 4g, 4h, 4i, 5b, 5d, 5e, 5g, and Supplementary Figures 3b, 4d, 5a.
